# Antimicrobial Applications of Clay Nanotube-Based Composites

**DOI:** 10.3390/nano9050708

**Published:** 2019-05-07

**Authors:** Anna Stavitskaya, Svetlana Batasheva, Vladimir Vinokurov, Gölnur Fakhrullina, Vadim Sangarov, Yuri Lvov, Rawil Fakhrullin

**Affiliations:** 1 Functional Aluminosilicate Nanomaterials Lab, Gubkin University, 119991 Moscow, Russian Federation; stavitsko@mail.ru (A.S.); vladimir@vinokurov.me (V.V.); namaz1000@gmail.com (G.F.); vs@asugubkin.ru (V.S.); 2 Bionanotechnology Lab, Institute of Fundamental Medicine and Biology, Kazan Federal University, 420008 Kazan, Republic of Tatarstan, Russian Federation; svbatasheva@gmail.com; 3 Institute for Micromanufacturing, Louisiana Tech University, Ruston, LA 71270, USA; ylvov@coes.latech.edu

**Keywords:** antimicrobial composites, halloysite, clay nanotubes, biofouling, microbiology

## Abstract

Halloysite nanotubes with different outer surface/inner lumen chemistry (SiO_2_/Al_2_O_3_) are natural objects with a 50 nm diameter hollow cylindrical structure, which are able to carry functional compounds both inside and outside. They are promising for biological applications where their drug loading capacity combined with a low toxicity ensures the safe interaction of these nanomaterials with living cells. In this paper, the antimicrobial properties of the clay nanotube-based composites are reviewed, including applications in microbe-resistant biocidal textile, paints, filters, and medical formulations (wound dressings, drug delivery systems, antiseptic sprays, and tissue engineering scaffolds). Though halloysite-based antimicrobial materials have been widely investigated, their application in medicine needs clinical studies. This review suggests the scalable antimicrobial nano/micro composites based on natural tubule clays and outlines research and development perspectives in the field.

## 1. Introduction

Antimicrobial agents have abundant formulations, found in various applications in food packaging, medical equipment, fabrics, polymers, paints, cosmetics, and even food. The production of materials with antibacterial properties is growing year by year. Industries need antimicrobials to ensure the safety of products and to prevent infections [1]. Together with the use of antibiotics for agricultural productions, the food industry demands new packaging materials with microbe-resistant properties, to guarantee the quality of products for a longer time. The fabrication of sustainable antimicrobial materials for medicine is crucially important, because of a growing number of hospital-acquired infections worldwide [2,3,4,5]. Removing microbes from the surfaces of medical equipment is challenging because of the microbial biofilm formation and complex multi- microbial systems allowing for the survival of persistent bacteria, even in the presence of antibiotics [6]. Infectious diseases associated with the formation of microbial biofilms on implantable medical devices seriously threaten patients and require recurrent operations in order to replace the infected device [7], making prevention of biofilm formation a high priority task.

The development of novel and long-lasting antibacterial materials should be performed in a careful and intelligent way. Currently, uncontrolled overuse of antibiotics has led to their accumulation in the environment and the appearance of new species resistant to antibiotic treatment [8,9,10,11,12,13]. Pharmaceuticals such as amoxicillin, erythromycin, triclosan, trimethoprim, and sulfamethoxazole are commonly detected in surface waters and soil [14]. The big challenge is the efficient disinfection of wastewater from farms and chicken plants, where a high organic content catalyzes bacterial growth and biofilm formations.

Therefore, the development of efficient and eco-friendly antimicrobial materials, which allow a decrease in uncontrolled antibiotic application while working for a longer time, is urgently needed. Clay minerals have been used in healing since early history. Aluminosilicates such as kaolinite, montmorillonite, and sepiolite are widely applied in industries, their properties and antibacterial composites based on this are reviewed in [15]. Iron-rich forms of clays (smectite, illite, etc.) are known to be active against human bacterial pathogens, and even drug resistant [16,17]. Chemical structures of clays such as imogolite (Al/Si-nanotube) make it antibacterial, due to the sorption of metals such as Fe, Co, Cu, and Zn, and its inner Al-rich surface [18]. Here, we cover the development and applications of antimicrobial materials using naturally-occurring tubular nanoclays. Halloysite clay nanotubes serve as a delivery vehicle for loaded bioactive compounds, helping to reduce the amount of antibiotics used by their controlled time-extended release.

**Figure 1:**
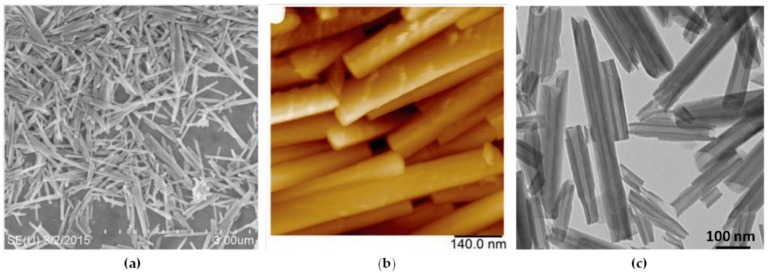
(**a**) Scanning electron microscopy (TEM), (**b**) atomic force microscopy (AFM), (**c**) transmission electron microscopy (TEM) images of halloysite. Reproduced with permission from [21], Copyright John Wiley and Sons, 2016.

Nanosized tubular halloysite (also termed halloysite nanotubes (HNTs)) is a dioctahedral 1:1 clay mineral of the kaolin group [19]. Its tubular structure was confirmed with transmission electron microscopy (TEM) and scanning electron microscopy (SEM). This multilayer tubular structure results in the rolling of aluminosilicate sheets under favourable geological conditions [19]. Transmission and scanning electron microscopy and atomic force microscopy images of halloysite are shown in Figure 1. Large halloysite deposits, allowing for the supply of hundreds of tons of the pure product, are found in Australia, the United States, China, New Zealand, and Turkey, although small amounts of halloysite nanotubes may be found at any kaolin deposit [20,21]. Halloysite tubes vary in length from 0.4 to 2 µm, in external diameter from 40 to 100 nm, and in internal diameter from 10 to 40 nm, but commercially available are 40–60 nm diameter and 0.5–1 µm length nanotubes [21,22,23].

The long history of human exposure to kaolin-type clay materials suggests the low cytotoxicity and high biocompatibility of these natural nanotubes, which was later proven in numerous experiments. Halloysite was found to be nontoxic for mammalian cells [24,25], nematodes [26], protozoa [27], bacterial [28], and yeast [29] cells, and was also tested as a food additive for mice, chicken, and piglets [17]. The nanosafety of halloysite favours its applications in materials intended to be in contact with humans and other organisms.

The chemical structure of halloysite, with a negative external surface composed of Si–O–Si and Si–OH and a positive internal surface of Al_2_O_3_ and Al–OH (at pH up to 8.5), makes it an appropriate template for the adsorption of both positively and negatively charged molecules and nanoparticles.

Halloysite is a potent candidate for the development of antibacterial coatings and paints, due to its hollow structure which is capable of encasing antiseptics and tuneable surface properties. One of the most convenient features of the clay nanotubes is that they do not need expensive and high energy consuming processes of exfoliation. Halloysites do not stack one to another like plates of kaolin, bentonite, or montmorillonite, and may be used as a powder, or an aqueous or paint dispersion. A number of reports are focused on producing polymer films and membranes with antibacterial compounds loaded inside halloysites [17].

Apparently, the idea of antibiotics encapsulation into clay nanotubes was proposed by Price, and Lvov, et al., [30,31,32], and these pioneering publications were followed by many others. The nanotube loading required mixing clay with a drug solution in water, alcohol, or acetone, taken at the highest concentrations, followed by the application of vacuum/air cycles and water washing at the end to remove the externally adsorbed molecules. In some cases, loading was performed from the drug melt. This process allowed for loading the nanotube lumen with 10–15 wt % of antibiotics or other drugs. Such formulations could be stored in dry state for years, and allowed for slow drug release within 10–20 h when placed in water. Additional coating of the loaded halloysites with polymers (e.g., dextran) or their embedding in polymers allowed the extension of the release to weeks and months.

One of the most promising applications of clay nanotubes is drug delivery. It is now generally accepted that rod-shaped nanoparticles are internalized in cells more efficiently than spherical ones [33,34,35]. The encapsulation of antibiotics into nanotubes allows for localized drug delivery, and limits undesirable side effects while ensuring the protection of sensitive drugs against oxidative or strongly acidic in vivo environments [36]. Functionalised nanotubes might be able to target specific cells, become ingested, and then release their contents in response to a chemical trigger [36,37]. This makes them promising for the creation of composite materials for bone [38,39] and tissue engineering [40,41], drug immobilization, and target delivery [42,43,44,45]. Several drugs were used in halloysite encapsulation: antibiotics—khellin, oxytetracycline, gentamicin, ciprofloxacin, vancomycin, atorvastatin, and metronidazole; and antiseptics—povidone iodine, amoxicillin, Brilliant green, chlorhexidine, salicylic acid, as well as dexamethasone, doxorubicin, furosemide, nifedipine, curcumin, resveratrol, and others. Novel drug delivery systems based on halloysite or its composite materials were reported, with an emphasis on topical formulations [21,46,47,48]. The only limitation for halloysite antibacterial composite applications in medicine is the restriction on intravenous injections due to slow nanoclay biodegradation and a lack of data on the halloysite injection influence on organisms.

## 2. Clay Nanotubes Loading with Antimicrobial Agents

### 2.1. Loading of Organic Compouds Inside Clay Nanotubes

**Figure 2:**
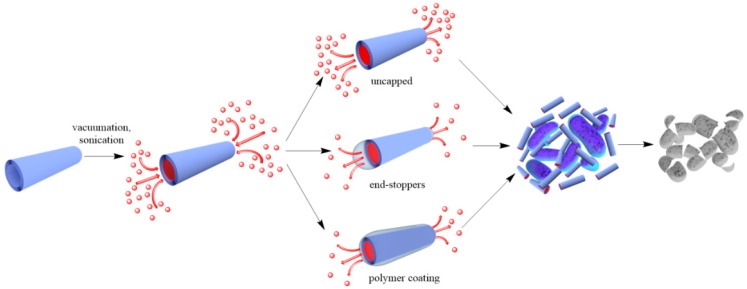
A scheme of antibacterial compound loading into halloysite lumens.

Two approaches are generally accepted to impart antibacterial properties to halloysite nanotubes: (a) loading of organic molecules inside halloysite lumen and adsorption on the external surface (Figure 2), and (b) outside grafting (Figure 2).

**Figure 3:**
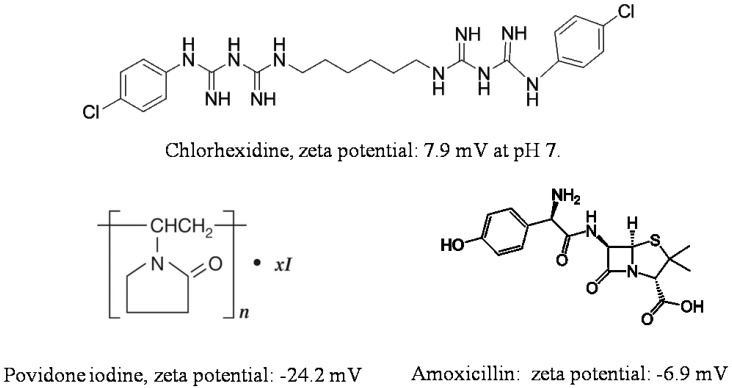
Chemical structure and zeta potential at pH 7 of commonly used antibacterial compounds.

Adsorption on halloysite and loading functional agents inside the nanotubes may be achieved by their stirring in drug solutions, assisted with sonication and vacuuming. The loading efficiency depends on the procedure and the charge and size of the molecule. Widely utilized chemicals such as chlorhexidine, povidone iodine, brilliant green, iodine, doxycyclin, amoxicillin, vancomicin, potassium calvulanate, gentamicin sulfate, and tetracyclin were loaded inside halloysite using sonication and vacuum [38,49,50,51,52,53]. Drug loaded halloysite was applied to produce antibacterial composites for bone regeneration, alginate-based wound dressing, electrospun membrane, and nanofibers [54,55,56,57]. Drug release kinetics is the most important parameter for long lasting antibacterial properties. The most practical and common anti-bacterial drugs are cationic chlorhexidine, anionic povidone iodine, and amoxicillin, the chemical structures of which are shown in Figure 3.

An investigation of chlorhexidine gluconate loading inside halloysite was performed in [58]. Though chlorhexidine gluconate has a positive charge, it was successfully loaded inside the nanotubes. The obtained composite showed a sustained drug release with a dialysis bag method; only 25% of chlorhexidine gluconate was released from the nanotubes within the first hour. By contrast, within the first hour, 75% of the unloaded drug was released from its untreated micropowder. Chlorhexidine gluconate loaded inside nanotubes was introduced into cotton fabric coating, to improve its antibacterial properties without the use of silver particles [59]. After coating, the fabric exhibited over 98% bacterial reduction for *Staphylococcus aureus*, *Escherichia coli*, and *Pseudomonas aeruginosa*. Even after 20 washes it showed 90% antibacterial activity, presumably due to a slow release of chlorhexidine gluconate from the nanotubes.

**Figure 4:**
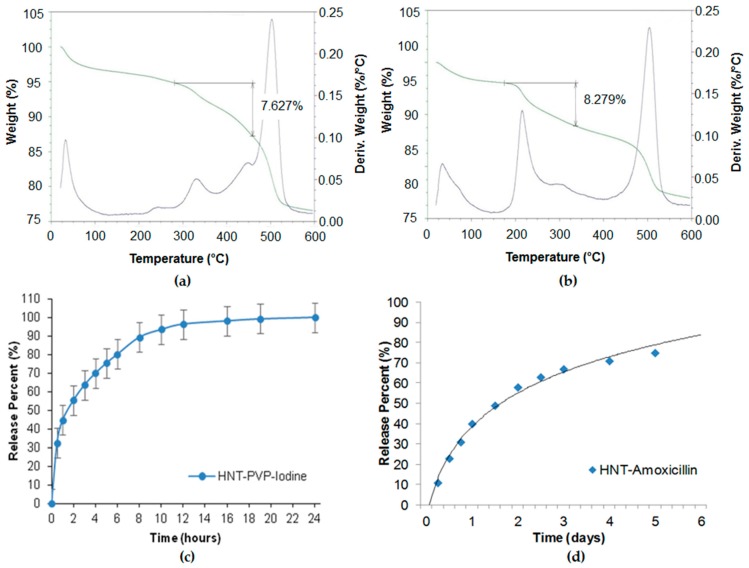
Thermogravimetric analysis (TGA) profiles of (**a**) povidone-iodine and (**b**) amoxicillin in halloysite, showing 7.6 and 8.3 wt % loading. Sustained release profiles of (**c**) povidone iodine and (**d**) amoxicillin from halloysite clay.

Povidone-iodine and amoxicillin have also been investigated for slower drug release [49]. In our study, we used saturated solutions of povidone iodine (80 mg/mL in water) and amoxicillin (50 mg/mL in ammonium hydroxide). Halloysite was added to the drug solutions and stirred to obtain homogeneous suspensions. The suspensions were placed in a vacuum for three cycles, 1 h each, to ensure maximum loading. Then the halloysite was washed twice to remove any excess/external drug, dried in a vacuum desiccator overnight, and powdered. Such powder formulations may be stored for a long time and dispersed in water with mechanical stirring before usage. Drug loading efficiency was determined by thermogravimetric analysis (TGA), as shown in Figure 4a,b. The drug release was analysed with a UV-Vis spectrophotometer at 224 nm (povidone iodine), and 227 nm (amoxicillin). The TGA curves showed 7.6 and 8.3 wt % of povidone-iodine and amoxicillin loading in halloysite. The loading could be increased with lumen enlarging by inner alumina etching in sulfuric acid. The release kinetics (Figure 4c,d) show the antiseptic extended release during 6–20 h. The formulations were efficient for the inhibition of *S. aureus* and *E. coli* for over one week.

Such formulations are also perspective for sprays, when clay nanotubes loaded with antiseptics are used in 3–5 wt % aqueous suspension and after being sprayed form thin coatings with prolonged antimicrobial activity.

The halloysite/salicylic acid system resulted in stable water suspension with a slow release of salicylic acid (over 50 h) [60]. The salicylic acid released from clay tubes showed an antibacterial activity at lower concentrations than free salicylic acid, likely due to the close contact or penetration of halloysite nanotubes in bacteria.

The efficiency reached 15 wt % when vancomycin was loaded in halloysite from a saturated solution [52]. The released drug exhibited high antibacterial activity to *S. aureus* and *B. streptococcus,* even after the long period of 33 days. The obtained system is proposed for bone and soft tissue infections treatment, where local antibiotic effect is needed.

Halloysite could be employed as a protecting carrier for chemically sensitive drugs and molecules. Ciprofloxacin attached to an APTES-functionalized halloysite helped to prevent complex formation with iron and other metal ions which are in contact with ciprofloxacin during drug delivery. The complexation studies between ciprofloxacin and iron revealed a 71 ± 1% decrease in undesirable drug absorbance [61]. Clay nanotubes were also investigated for the loading of volatile compounds, like essential oils, to prevent them from evaporation and losing antibacterial activity [62,63,64,65,66].

**Figure 5:**
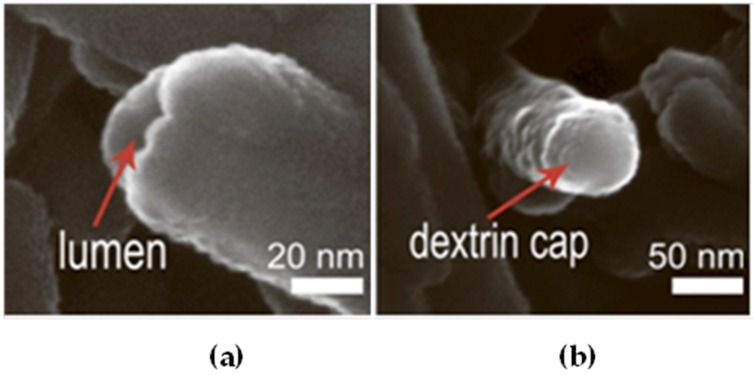
SEM images of (**a**) an open halloysite lumen, (**b**) a dextrin capped halloysite lumen. Reproduced from [67] under Creative Commons Attribution 4.0 International License.

To reduce the release rate of drug molecules from clay nanotubes, the end-stoppers or polymer surface coating could be synthesized. Dextrin caps were formed on the nanotubes end to prevent early drug release (Figure 5) [67]. Benzotriazole–copper coating on halloysite nanotubes provided a more sustained release of brilliant green, extending it from 50 to 200 h [50]. In [65], thyme oil was encapsulated inside clay nanotubes and then caged inside using end-stoppers or surface coating. Both ends of the nanocapsules were capped with a sodium alginate-calcium chloride complex. Coating the negatively charged surface of untreated clay nanotubes was performed using the layer-by-layer method with positively charged polyethyleneimine. The surface coating of halloysite significantly extended the release behaviour of essential oils at room temperature over that of untreated and end-capped nanotubes.

**Figure 6:**
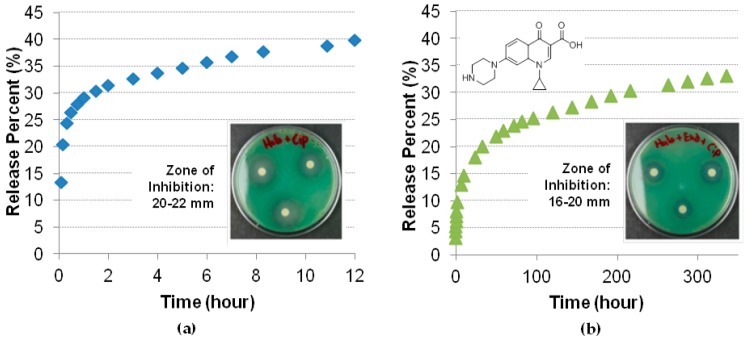
Ciprofloxacin sustained release (**a**) from halloysite tubes and *P. aeruginosa* inhibition; (**b**) from 8% halloysite composite with polymethmetacrylate bone cement and *P. aeruginosa* 48 h inhibition. Reproduced with permission from [21], Copyright John Wiley and Sons, 2016.

A standard zone of inhibition assay was developed to compare the antibacterial action of the ciprofloxacin in solution and encapsulated in halloysite; in all cases, 5 µg of the antibiotic was loaded [21]. The size of the zone of inhibition indicates the effectiveness of the antibiotic and is often used as a quick test for the resistance of a strain. After each 24 h, the ciprofloxacin loaded discs were moved and added to a new spread plate to get a new zone of inhibition. Ciprofloxacin not loaded in the nanotubes worked only within 12 h. The nanotube formulations were effective over four days (Figure 6). This indicates a prolonged release that remains effective against multidrug resistant *P. aeruginosa* over an extended time. Even longer efficiency was observed when ciprofloxacin loaded halloysite was composited with bone implant cement polymethmetacrylate.

The exponential increase in bacterial resistance to chemical antibiotics and losing their effectiveness in the treatment of infections initiated the interest of researchers and pharmaceutical industries to the application of peptides as therapeutic antimicrobial agents [68]. Nicin and pediocin were successfully loaded inside halloysite [69]. The antimicrobial activity was better when halloysite nanotubes were used as a support agent rather than octadecylamine-modified montmorillonite or bentonite clays.

### 2.2. Grafting of Antimicrobial Nanoparticles on Clay Nanotubes Surfaces

**Figure 7:**
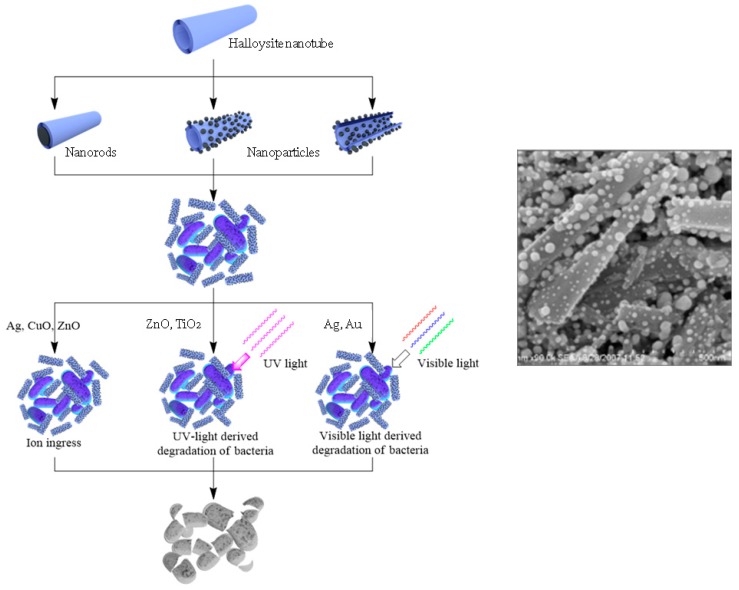
Scheme of antibacterial metal structures deposition onto/into the nanotubes, with their antibacterial applications and image of silver particles coated onto halloysite.

The loading of organic antiseptics inside halloysite, with its further slow release, may have a limitation. It should be taken into consideration that the exposure of pathogens to progressively lower drug concentrations could become a risk factor for the selection of drug resistant strains. This is why other strategies should be developed. A possible issue lies in producing antibacterial composites with metals. Nanostructures containing silver, copper, zinc, iron oxides, and their combinations are well known for killing even antibiotic resistant bacteria, which is why they are widely applied for making materials with antibacterial properties [70,71,72,73,74,75]. The use of templates helps to overcome the aggregation of particles and their release to the environment. Halloysite has great potential as a carrier for metal particles grafting and metal complexes formation [76,77]. Possible metal/halloysite structures are shown in Figure 7, together with various mechanisms of killing pathogenic species.

Silver is a well-known antimicrobial metal. The vacuum loading and grafting of silver on/in modified clay nanotubes are common procedures, starting from [74].

Ag-nanorods were also synthesized inside the lumen of halloysite by thermal decomposition of silver acetate loaded from an aqueous solution [78]. Encasing silver inside the nanotubes allowed the prevention of colour changes in Ag-halloysite doped white paint, while the direct addition of silver nanoparticles converted the coating to a yellow-grey color. In [79], the dispersion of halloysite in a silver salt solution was kept in a vacuum for five cycles, followed by reduction with curcumin. Curcumin is not only a reducing agent, but it also possesses antibacterial activity against gram-positive and gram-negative bacteria. To enhance loading efficiency, an acid treatment of halloysite was used, creating new sites for metal cluster formation inside the tube’s lumen [80].

Modification of clay outer surfaces is another efficient way to decorate them with nanoparticles. In order to get the modified halloysite nanotubes loaded with silver nanoparticles, a series of reactions—including in situ polymerization—were suggested [81]. First, [3-(2-aminoethyl)aminopropyl trimethoxysilane (KH-792 silane) was bound to halloysite, followed by double-bond grafting using acryloyl chloride. Then poly(4-vinylpyridine) was formed on the surface using in-situ polymerization, with a final Ag attachment. A similar approach was proposed for Ag nanoparticles, where KH-792 silane was used as a complexation agent for ions [82]. The average diameter of particles formed after reduction was 5 nm. These materials showed good antibacterial activity against gram-negative bacteria (*E. coli*) and gram-positive bacteria (*S. aureus*).

In [83], chitosan was used to immobilize silver nanoparticles on halloysite because it is a good complexation agent that contains nitrogen atoms for binding silver ions through electron pair sharing. Chitosan helps to prevent the leaching of silver ions into the media. To graft chitosan, the surface of the halloysite was modified with 1,6-hexamethylene diisocyanate. Silver nitrate was used, and after the reduction of the silver–chitozan complex, silver nanoparticles of about 5 nm were observed on the tube’s surface. It is also possible to produce silver particles by wet impregnation on aminosilane-modified halloysite [84]; though the particle size distribution is broad and not many particles are attached, this procedure is one of the simplest.

In [72], ZnO nanoparticles were deposited on the outer and inner surfaces of halloysite nanotubes using a solvo-thermal method, and then incorporated into a polylactic acid matrix to give an antimicrobial membrane. Zinc oxide particle formation on clay tubular templates was also investigated in [85]. ZnO-Ag/halloysite composites for antibacterial applications were synthesized using thermal decomposition of zinc salt, followed by the reduction of the silver ions with NaBH_4_ [86]. CuO, TiO_2_, and Au nanoparticles were synthesised, exploiting halloysite surfaces as templates for the formation of metal nanoparticles and nanoclusters with tuneable properties [73].

## 3. Surfaces and Liquids Disinfection and Protection Using Clay Nanotubes-Based Antimicrobial Nanocomposites

One of the biggest problems in medical facilities is the growing number of hospital acquired infections. The World Health Organization reported that one of the most common routes for transmission of infectious diseases is by indirect contact with surfaces contaminated with infectious droplets produced by patients’ coughing, sneezing, and talking. Many microbes and viruses can survive for days on surfaces. Hand contact with contaminated surfaces (i.e., fomites) and subsequent transfer of microbes to the mucosal membranes of the mouth, nose, and eyes is the cause of many reported gastroenteritis outbreaks and other infections [87]. The efficacy of antimicrobial agents is seriously threatened by an alarming increase in microbial resistance. In 2016, the United Nations General Assembly adopted a political declaration giving full attention to antimicrobial resistance, following a call for global action by the World Health Organization. It has been recognized that rates of antibiotic resistance among bacteria are higher in developed nations, particularly in the US and Europe, which hinders long-time disinfection with standard methods, like wiping surfaces with disinfection solutions [88,89]. Formulations with sustained antiseptic delivery could be more efficient than today’s simple sprays like aqueous chlorine dioxide. In this regard, a number of long-lasting antimicrobial systems have been reported, including biocidal nanomaterials such as silver nanoparticles, light-activated photocatalysts based on TiO_2_, surface-tethered bactericides quaternary ammonium compounds, and phosphonium salts. These materials are effective against a wide spectrum of microorganisms. Halloysite loaded with drugs, or decorated with nanoparticles and compounded with paints, or incorporated into hydrophobic surface coating could become a good alternative to traditional disinfection procedures. Halloysite’s zeta-potential is ca. −30 mV, which does not allow for long stable aqueous dispersions, however, in many cases, internal nanotube loading with anionic drugs drastically increases the halloysite zeta-potential to −50–60 mV, making such formation of stable water-based dispersions applicable for convenient antibacterial sprays that are easily applied to surfaces.

Photo-induced degradation of bacteria using photocatalysts has been widely investigated for water purification. Halloysite nanotubes were applied as a support for photocatalytic nanoparticles, where they act as a stabilizer to prevent the aggregation of nanoparticles [90,91,92,93,94]. One of the most commonly used photocatalysts is TiO_2_ and its nanocomposites [95,96,97,98]. TiO_2_ only absorbs wavelengths in the near-UV region (λ < 400 nm), which is about 3% of the solar spectrum, and it cannot efficiently utilize visible light, which is about 43% of the solar spectrum, for photocatalytic disinfection [95]. Its modification may change the band gap and make it more efficient in visible light [98,99,100]. Photocatalytic systems based on halloysite and TiO_2_ were tested mainly for the degradation of pollutants and as a photocatalyst, but it could be used for antimicrobial applications too [93]. Antibacterial ZnO nanoparticles also act as photocatalysts in UV-light. In [85], ZnO nanoparticles adsorbed on halloysite were developed as a UV barrier for bacteria formation on membranes.

In [84], a role of the plasmonic excitation of silver nanoparticles decorating halloysite nanotubes in their bioactive properties is discussed. The optical absorption measurement revealed a broad plasmonic resonance in the region of 400–600 nm for halloysite with silver nanoparticles. The later samples exhibited a bactericidal effect, which is more pronounced under illumination. Therefore, Ag-functionalized clay seems to be promising for antibacterial treatments of liquids and surfaces stimulated by visible light exposure. Gold-nanoparticles could be applied in the same manner due to plasmonic effects. Halloysite-based core-shell structures with gold coating showed that the morphology, interconnectivity, and thickness of the Au shell define the optical response and photothermal capacity of plasmonic materials [101,102].

## 4. Application of Clay Nanotube-Based Antibacterial Composites

### 4.1. Bone and Tissue Engineering

**Figure 8:**
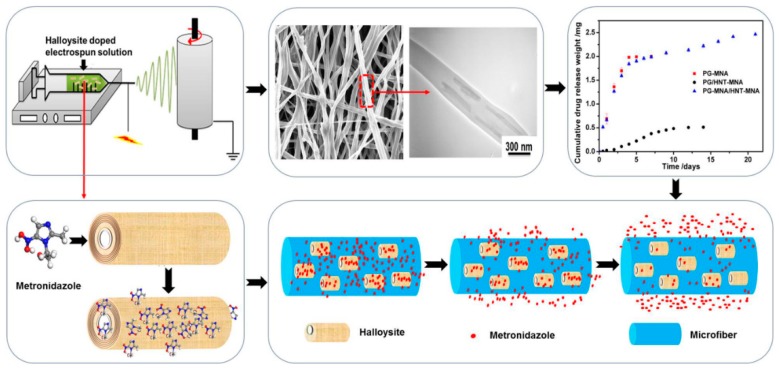
Electrospinning drug-loaded halloysite clay nanotubes doped into poly(caprolactone)/gelatin microfibers. Reproduced with permission from [104], Copyright American Chemical Society, 2015.

Various functional materials containing halloysite loaded with antibacterial particles or drugs for medical application, coatings, and polymeric films have been developed recently. Nanofibers made with an electrospinning technique have been extensively studied for tissue engineering and other applications. Drug loaded fibers are not only biocompatible due to the polymers used, but also have antibacterial properties with slow drug release due to polymer degradation. Various halloysite-polymer composite fibers made using polylactic acid [54,103], polycaprolactone [49], poly(caprolactone)/gelatin [104] (Figure 8), and poly(lactic-co-glycolic acid) [57] were studied for antimicrobial protection.

In [57], electrospun poly(lactic-co-glycolic acid) nanofiber was modified with halloysite to make a drug delivery system with slow release of tetracycline hydrochloride. The drug loading efficiency was 42.65%. Halloysite loading leads to lower fiber diameter caused by the incorporation of positively charged tetracycline hydrochloride into the electrospinning solution. The tetracycline hydrochloride/halloysite/poly(lactic-co-glycolic acid) nanofibrous mats showed good cytocompatibility, and displayed effective antibacterial activity and ability to inhibit bacterial growth both in liquid and on solid mediums. The release percentages for nanofibrous mats with drug loaded halloysite during the 42 days were much lower than those of the nanofibers with pure drug and tetracycline hydrochloride/halloysite powder within the first day.

Guided tissue/bone regeneration membranes with sustained drug delivery were developed by electrospinning drug-loaded halloysite clay nanotubes doped into poly(caprolactone)/gelatin microfibers [104]. The use of 20 wt % nanotubes in fiber membranes allowed for 25 wt% metronidazole drug loading in the membrane.

Halloysite nanocontainers were used to obtain new dental materials with bioactive and antimicrobial properties, in order to improve clinical outcome in daily practice [105,106,107]. In [108], halloysite plays the role of a drug delivery system for dental implant coatings. The vancomycin loaded HNTs/chitosan composite coatings were electrophoretically deposited (EPD) from different alcoholic suspensions. Ethanol suspensions were selected as the optimum because of their high EPD rate, as well as the formation of coatings with relatively high roughness and high contents of adsorbed and non-adsorbed chitosan. The coating was characterized by burst release during the initial hours (3 h) after immersion in phosphate-buffered saline PBS and then long term slow release. Drug loaded coating showed higher antibacterial activity (about 27%) against *S. aureus* compared to the similar coating without loaded drugs.

### 4.2. Wound Dressing

Halloysite can be applied for making flexible multi-layer wound dressings that possess tuneable functionalities, including fluid absorption, antibacterial/fungal protection, and tissue regeneration. The dressing could be used for both prophylactic and therapeutic interventions, as a wound packing material, or as a topical gauze or pad. The addition of doped clay nanotubes provides enhanced dressing properties, the potential to load multiple drug sets, and increased control over the drug release kinetics (50+ hours, all these are favorable properties for the treatment of chronic unhealing wounds, multiple microbial infections, and for multi-vector treatments [49].

A dual drug co-delivery with elastic antibacterial nanocomposite was developed by combining ciprofloxacin and polymyxin B sulfate-loaded halloysite nanotubes into a gelatin elastomer. Ciprofloxacin nanoparticles which act against both gram-positive and gram-negative bacteria were dispersed directly in the matrix, and polymyxin B sulfate was loaded into halloysite and then distributed into the matrix. The effect of ciprofloxacin and polymyxin-loaded halloysite formulations on physical properties, cytotoxicity, fibroblast adhesion and proliferation, in vitro drug release, and antibacterial properties were investigated. Drug loaded halloysite not only enhanced the matrix tensile strength but also slowed down the release rate of the high dissoluble polymyxin B sulfate [109].

### 4.3. Filtration Membranes with Enhanced Antibacterial Activity

**Figure 9:**
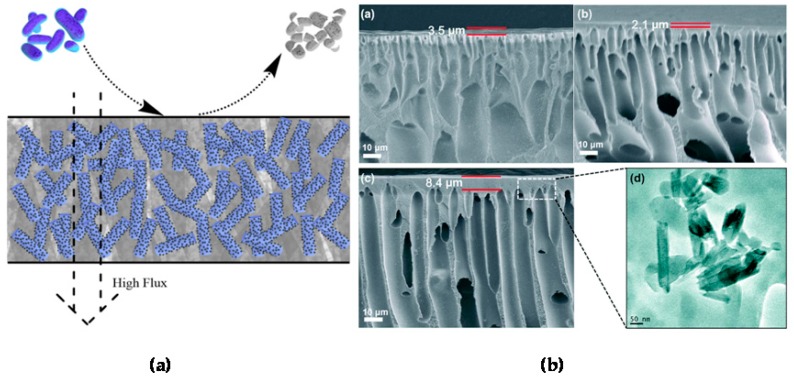
(**a**) General scheme of a filtration membrane with antibacterial halloysite based fillers. (**b**) SEM images of the cross-section morphology of membranes: (**a**) pure polyethersulfone membrane, (**b**) polyethersulfone membrane containing 1% *N*-halamine@halloysite, and (**c**) polyethersulfone membrane containing 3% *N*-halamine@ halloysite; (**d**) TEM image of the presence of N-halamine@halloysite in the polyethersulfone hybrid membrane. Reproduced with permission from [113], Royal Society of Chemistry, 2015.

Filtration membranes doped with halloysite have been reported (Figure 9a). Membranes working in water, especially with high organic content, are hindered by biofouling, which decreases the membrane lifetime and increases energy consumption [110]. The inclusion of nanotubes functionalized with antibacterial agents helps to inhibit the development of biofilms. Halloysite was used for membrane preparation, allowing for sustained antimicrobial properties and preventing bio-contamination [72].

N-halamine@halloysite nanocomposites were synthesized and added at 1–3 wt% to a polyethersulfone filter membrane (Figure 9b) [107]. The hybrid membrane was thermos-mechanically stable, had lower surface roughness than the pure polyethersulfone membrane, and the water flux had been increased. The hydrophilicity of the membrane was also enhanced by the addition of halloysite. Immobilization of enzymes serving as natural antibacterial agents on clay nanotubes is another way to face the biofilm problem. Lysozyme was covalently grafted to halloysite functionalized with carboxylic groups, and was then added to polyethersulfone to prepare hybrid antibacterial ultrafiltration membranes [111]. The antibacterial tests revealed that hybrid halloysite membranes demonstrated a good antibacterial performance and were applicable for wastewater treatment.

Polyethersulfone ultrafiltration membranes bearing modified halloysite loaded with A- nanoparticles [81] and copper ions [112] were prepared via phase inversion. The Ag-nanoparticles were grafted as described earlier and the Cu^2+^-halloysite was synthesized by chemical modification of halloysite with silane, and then mixed with copper dichloride for complexing the Cu- ions. Tests showed that the hybrid membranes had a good antibacterial property, and the protective rates against *E. coli* and *S. aureus* were more than 99%.

### 4.4. Food Contact Materials

Food package materials like polymeric films are susceptible to bacteria colonization and biofilm formation. Meat, fish, cheese, bread, fruits, and vegetables are especially sensitive to bacteria and fungi. The development of polymer composites with antibacterial fillers preventing food from decay, and microorganism growth is very important. During recent years, various types of antimicrobial agents such as essential oils, plant extracts, and natural polymers have been tested in food packaging because of their minimal environmental impact and biodegradability [65,114,115]. Essential oils derived from plants are widely known for their activity, but they are difficult to incorporate into polymers due to their volatility.

Recent studies demonstrated that halloysite nanotubes can be used as active carriers, retaining essential oils during the high-temperature compounding of polymers and preserving high antimicrobial properties for a required time. Low-density polyethylene films containing carvacrol/halloysite showed sustained anti-microbial properties against *E. coli* and *L. innocua*, as well as fungicidal activity against *A. alternata* [64]. Carvacrol and thymol mixtures adsorbed on halloysite showed synergetic antibacterial effects after incorporation into an low-density polyethylene film intended for food package [63]. Carvacrol loaded inside nanotubes was incorporated into low-density polyethylene and ethylene vinyl alcohol copolymer films [62]. Multi-layered low-density polyethylene/ethylene vinyl alcohol copolymer films with loaded halloysite produced by a forced co-extrusion process exhibited a high carvacrol content despite the harsh conditions (200 °C and a long treatment time). Nanocomposites based on pectin-halloysite hybrids with rosemary oil also showed good potential for packaging [66]. Packaging paper containing thyme oil loaded halloysite exhibited strong antibacterial activity against *E. coli* for 10 days [65].

Table 1 shows a summary of antibacterial organic molecules loaded inside halloysite nanotubes using different procedures. The release kinetics of various agents from the organic–inorganic hybrids and composites based on loaded clay nanotubes are presented.

## 5. Conclusions

The loading of antibacterial chemicals inside natural halloysite clay nanotubes and capping them with end-stoppers or coatings allow for materials with slow release kinetics (days and weeks) and long-lasting effects. Due to high dispersibility and SiO_2_-surface chemistry, these nanotubes are a promising filler for composite materials with polymeric films and fibers, allowing for bacterial protection due to antiseptic release. Such nanocomposite formulations could be used for the preparation of wound dressings, fabric coatings, fibers for tissue engineering, and dental and suture materials. A very interesting and simple solution could be antibacterial sprays with water dispersions of halloysite loaded with antiseptics. These clay nanotubes are good candidates for drug delivery systems, especially when a topical effect is needed. Antibacterial halloysite formulations are currently tested, mostly in laboratories and their medical applications need more intensive clinical studies. One of the more significant deterrents limiting the use of such clay-based antimicrobial composites in clinical studies is the lack of cooperation between scientists working in the area of materials and composites preparation and in medical fields. To tackle this challenging issue, the communication between material science, medical science, and medical practices should be facilitated.

## Figures and Tables

**Table 1:** Loading efficiency and release kinetics of organic antibacterial agents loaded inside halloysite nanotubes.

№	Anticeptic	Chemical Formula, Molecular Mass (g/mol.)	Loading Procedure	Loading Efficiency, wt %	Release Kinetics	Application	Reference
1	Gentamicin	C_21_H_43_N_5_O_7_, 477.6	Vacuum cycling, washing	11	94% after 48 h (halloysite_gentamicin); 60% in 10.4 days (PMMA_ halloysite_gentamicin composite)	Bone cement	[38]
2	Brilliant Green	C_27_H_33_N_2_HO_4_S, 482.64	Vacuum cycling, washing	15–20	96% after 5 h, 99.9% after 1.1 day (Halloysite-PCL Scaffold); Cu-BTA coated BG/halloysite 99% after 8.3 days	poly-e-caprolactone scaffolds	[49,50]
3	Metronidazole	C_6_H_9_N_3_O_3_, 171.2	mixing	25	Metronidazole/halloysite 70% after 10 h; Polycaprolactone/gelatin polymer/Metronidazole/halloysite 90% after 15 days	anti-infective GTR/GBR implant membrane	[104]
4	Chlorhexidine	C_22_H_30_Cl_2_N_10_, 505.45	Vacuum cycling, washing	15–20	85% after 4 h	Scaffolds, wound repair, patient recovery.	[49]
	Chlorhexidine gluconate	C_34_H_54_Cl_2_N_10_O_14_, 897.76	Vacuum cycling, washing		25% after 1 h	Cotton fabric coating	[58]
5	Povidone iodine	C_6_H_9_I_2_NO, 364.95	Vacuum cycling, washing	15–20	76% after 6.5 h	Scaffolds, wound repair, patient recovery.	[49]
6	Doxycyclin	C22H24N2O8, 444,43	Vacuum cycling, washing	15–20	99% after 4 h	Scaffolds, wound repair, patient recovery.	[49]
7	Iodine	I_2_, 253,8		15–20	93% after 5 h	Scaffolds, wound repair, patient recovery.	[49]
8	Vancomycin	C_66_H_75_Cl_2_N_9_O_24_, 1449.3	Vacuum cycling + sonication, washing	15	50% at pH 7 after 1 day 74% at pH 7 after 5 weeks	local antibiotic delivery systems	[52]
9	Tetracycline base	C_22_H_24_N_2_O_8_, 444.4	vacuum cycling, two step loading	39	Hall coated with chitosan 80% after 16 days	Periodontitis treatment	[53]
	Tetracycline hydrochloride	C_22_H_25_ClN_2_O_8_, 480.9	vacuum cycling, two step loading	42,6	TCH/HNTs/89.4% after 24 h; TCH/HNTs/PLGA composite nanofibers 16–18% after 24 h; 68–76% after 42 days	drug-loaded electrospun nanofibers	[57]
10	Amoxicillin	C_16_H_19_N_3_O_5_S, 365,4	Vacuum cycling, washing		halloysite nanotubes/AMX 43% after 24 h; poly(lactic-co-glycolic acid)/halloysite g/mol,nanotubes/AMX/chitosan nanofibers 36% after 24 h	Wound healing	[56]
11	Salicylic acid	C_7_H_6_O_3_, 138,12	Vacuum cycling, washing	10.5	60% after 10 h; 100% after 50 h	Active packaging for food industry	[60]
12	Polymyxin B sulfate	C_56_H_100_N_16_O_17_S, 1301.57	Vacuum cycling, washing	13	gelatin-based nanocomposites 50% after 70 h		
13	Carvacrol	C_10_H_14_O, 150,22	Sonication	33	LDPE/(HNTs/carvacrol hybrid diffusion coefficient of 4.22 * 10^−11^ m^2^ s^−1^	Active packaging for food industry	[62,63,64]
14	Thyme oil (TO)	Variable mol. mass, Mixture of compounds	sonication	5–7	TO/HTNs on air 69% after 9 day; TO/capped HNTs 33% after 9 days; TO/polymer coated HNTs 28% after 9 days	Paining for food packaging	[65]
15	Rosemary essential oil	Variable mol. mass, Mixture of compounds	Vacuum cycling	~50	Nano-hybrid/pectin 25% after 4 h; 90% after 28 days	biodegradable materials for packaging	[66]
